# Gas surrounding the urinary bladder in emphysematous cystitis

**DOI:** 10.1590/S1677-5538.IBJU.2016.0555

**Published:** 2017

**Authors:** Zhenyu Yang, Chang Sheng

**Affiliations:** 1 Department of Urology, Pudong New Area People's Hospital, Shanghai, China

**Keywords:** Cystitis, Tomography, X-Ray Computed, Diabetes Mellitus

## Abstract

We report a rare case of emphysematous cystitis in a 66-year-old woman with a history of diabetes mellitus. The predisposition of diabetes mellitus and infection of gas-forming bacteria is considered to precede the manifestation of emphysematous cystitis. The present recommended diagnosis test is computed tomography, which have definite value in the evaluation of gas accumulation in bladder wall, or an air-fluid level in bladder.

## INTRODUCTION

**Figure 1:**
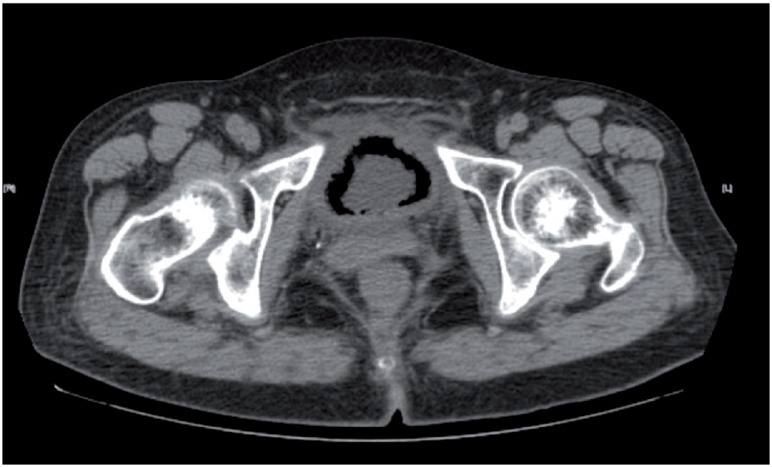
Gas surround the bladder wall on computed tomography.

**Figure 2:**
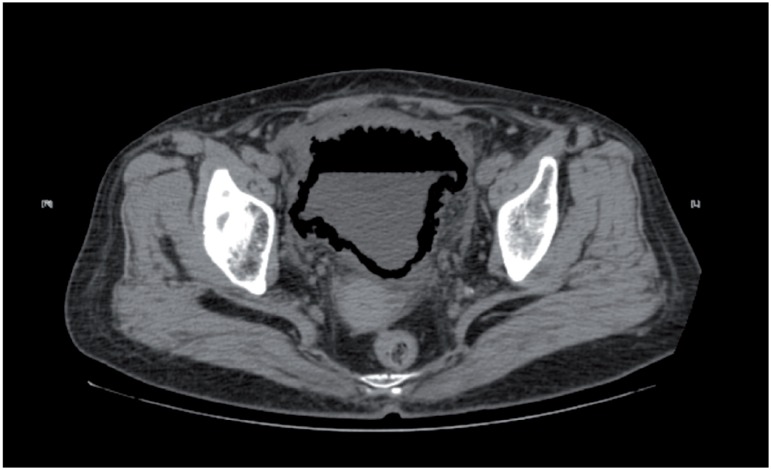
A prominent air-fluid level within the bladder on computed tomography.

A 66-year-old woman with a 10-year history of diabetes mellitus presented to the emergency department for painful urination and gross hematuria. Similar episodes had occurred several times in the 6 months preceding presentation, along with episodes of acute urinary retention and bladder catheterization. The physical examination was unremarkable. Laboratory investigations revealed mild anemia (Hb:9.2g/dL) and elevated blood glucose (BG:171mg/dL). Urinalysis findings indicated urinary tract infection. The culture of voided midstream urine showed evidence of Escherichia coli. Urinary system ultrasonography revealed an irregular thickened bladder wall with post-void residual volume of 140mL. Computed tomography (CT) of the abdomen and pelvis without administration of contrast material revealed diffuse gas within the bladder wall (Figure-1) and a prominent air-fluid level (Figure-2). This pattern of gas surrounding the urinary bladder on computed tomography is a typical manifestation of emphysematous cystitis (EC) in which natural fermentation of glucose for gas-forming bacteria infected mostly in diabetic women (1, 2).

EC is a rare, but severe infection of the bladder characterized by gas accumulation surrounding the bladder wall. It occurs predominantly in females over 60 years old, with 60-70% of cases being diabetic patients (1). Diabetes mellitus and female gender are the highest risks for developing EC. The typical presentation spectrum of EC includes lower abdominal pain, bacteremia, and dysuria. Urinalysis often indicates bacteriuria, pyuria and hematuria. CT is the most sensitive diagnostic protocol for EC (3). Current concepts about the pathogenesis of gas formation in the bladder is postulated that bacteria such as Escherichia coli. ferment the glucose in the urine of diabetic patients but in non-diabetic patients remains still unknown. EC is often successfully managed with drainage and appropriate antibiotics. About 10% of cases require surgery and estimated mortality rate is 7% (4, 5). Our patient was treated with levofloxacin 500mg for 5 days and was discharged in stable condition.
